# Weight Suppression, Binge Eating, and Purging as Predictors of Weight Gain During Inpatient Treatment in Persons With Bulimia Nervosa

**DOI:** 10.1002/erv.3197

**Published:** 2025-04-07

**Authors:** Adrian Meule, Anna L. Dieffenbacher, David R. Kolar, Ulrich Voderholzer

**Affiliations:** ^1^ Department of Psychology University of Regensburg Regensburg Germany; ^2^ Schoen Clinic Roseneck Prien am Chiemsee Germany; ^3^ Department of Psychiatry and Psychotherapy LMU University Hospital LMU Munich Munich Germany; ^4^ Department of Psychiatry and Psychotherapy University Hospital of Freiburg Freiburg Germany

**Keywords:** binge eating, bulimia nervosa, purging, weight gain, weight suppression

## Abstract

**Objective:**

Persons with bulimia nervosa (BN) often gain weight during treatment, which potentially poses a threat to treatment adherence. Although weight suppression has been found to be a predictor of weight gain in persons with BN, research about the trajectory of weight changes during treatment and other predictors thereof is scarce.

**Method:**

The current study examined weight suppression as well as self‐reported binge eating severity and purging frequency at admission as predictors of weight change in 746 persons with BN (95% female) who received inpatient treatment at the Schoen Clinic Roseneck (Prien am Chiemsee, Germany) between 2015 and 2020.

**Results:**

Body mass index (BMI) increased linearly across treatment weeks. Higher weight suppression predicted larger weight gain, particularly in those with a relatively low BMI at admission. More frequent purging and less severe binge eating predicted larger weight gain but high binge eating severity in combination with infrequent purging attenuated this effect.

**Conclusions:**

Results replicate that those with high weight suppression are at higher risk for gaining weight during BN treatment but extend these findings in that this effect additionally depends on current BMI, similar to findings reported in persons with anorexia nervosa. They further demonstrate that the core features of BN—binge eating and purging—also predict weight change both separately and interactively and may, therefore, be considered in psychoeducation and therapy planning.

**Summary**

- Persons with bulimia nervosa gained weight during inpatient treatment
- Weight suppression interacted with body mass index at admission when predicting weight change
- Self‐reported purging interacted with binge eating at admission when predicting weight change

## 1 Introduction and Aims

Most persons with bulimia nervosa (BN) have normal weight and, therefore, weight gain or weight loss is usually not an aim during BN treatment (Gendall et al. 1999). However, persons with BN show a small but statistically significant average weight gain during treatment (e.g., Hessler et al. 2018). Yet, little is known about the trajectory of weight changes during treatment in persons with BN. For example, while studies indicate that weight gain is non‐linear in persons with anorexia nervosa in that there is an attenuation of weight gain rate over time (Jennings et al. 2018; Meule et al. 2022; Vansteelandt et al. 2010), such studies about the kind of weight trajectories do not exist for BN. As fear of weight gain is one of the main drivers of purging behaviors, it may prevent people with BN to seek treatment in the first place (Ali et al. 2017; Regan et al. 2017) and, if they seek treatment, weight gain during treatment potentially poses a threat for treatment adherence (Juarascio et al. 2018). Thus, research that seeks to better understand factors that predict weight gain during treatment of BN can inform psychoeducation programs and better support those persons identified to be at greater risk of weight gain.

Research on predictors of weight change during treatment in persons with BN is scarce and most studies have largely focused on weight suppression as predictor variable. Weight suppression refers to the difference between a person's highest body weight at their current height and that person's current body weight (Lowe 1993). That is, a person with a high weight suppression has lost more weight sometime in their life than a person with a low weight suppression. Higher weight suppression has been found to predict larger future weight gain in persons without eating disorders (Lowe et al. 2006a, 2019; Meule and Platte 2018; Stice et al. 2011), persons with anorexia nervosa (Carter et al. 2015; Hessler et al. 2019; Kaufmann et al. 2021; Wildes and Marcus 2012), and persons with BN (Carter et al. 2008; Herzog et al. 2010; Hessler et al. 2018; Lowe, Davis, et al. 2006b) as well as smaller weight loss in persons with obesity (Call et al. 2019). In addition, some studies reported interactive effects in persons with anorexia nervosa and other samples such that effects of weight suppression on weight gain were stronger in persons with relatively low body mass index (BMI) at baseline (Berner et al. 2013; Call et al. 2021; Meule et al. 2022; Witt et al. 2014), but such an interactive effect has not yet been reported in persons with BN.

In the current study, we examined weight change and predictors thereof during inpatient treatment in persons with BN. Based on earlier reports (e.g., Hessler et al. 2018), we expected that BMI would significantly increase during treatment. As weight gain during inpatient treatment in persons with anorexia nervosa has been found to be non‐linear (Jennings et al. 2018; Meule et al. 2022; Vansteelandt et al. 2010), we explored whether the hypothesized weight gain would be linear or non‐linear across treatment weeks. Furthermore, we expected that higher weight suppression would predict larger weight gain, in line with earlier findings (Carter et al. 2008; Herzog et al. 2010; Hessler et al. 2018; Lowe, Davis, et al. 2006b). As weight suppression interacted with current BMI when predicting weight gain in persons with anorexia nervosa and other samples (Berner et al. 2013; Call et al. 2021; Meule et al. 2022; Witt et al. 2014), we explored whether such an interactive effect would also be found when predicting weight change in persons with BN.

Based on the scarcity of findings about predictors of weight change during treatment in persons with BN, we also tested two other potentially relevant predictor variables. Specifically, binge eating and purging (e.g., self‐induced vomiting) are the core features of bulimic symptomatology (American Psychiatric Association 2013; World Health Organization 2022). While more frequent binge eating has been found to predict weight gain in other populations (Masheb et al. 2015; Park et al. 2015; Sonneville et al. 2013), its role in persons with BN is less clear. For example, high binge eating severity may have already led to weight gain before treatment and, thus, may not be predictive of future weight gain (or may even be predictive of weight loss) when binge eating is terminated during treatment. In contrast, frequent purging might have induced larger weight loss before treatment and may, therefore, be predictive of larger weight gain during treatment when purging is terminated during treatment. Yet, binge eating episodes are not always followed by purging and purging is not always precipitated by binge eating (American Psychiatric Association 2013, 346; Núñez‐Navarro et al. 2011). Thus, based on the idea that binge eating and purging do not always co‐occur, they might interact when predicting weight change such that different combinations of high/low binge eating severity and frequent/infrequent purging may differentially predict weight change during treatment. Because of the exploratory nature of these research questions and the large sample size, we only considered effects as significant when *p* < 0.005, as has been suggested for new discoveries (Benjamin et al. 2018).

## 2 Method

### 2.1 Sample Description

According to the ethics committee of the LMU Munich, retrospective analyses of completely anonymized data are exempt from requiring ethical approval and informed consent. Data of 746 persons with BN (ICD–10 code F50.2) who were admitted to inpatient treatment at the Schoen Clinic Roseneck (Prien am Chiemsee, Germany) between 2015 and 2020 were analyzed. Mean age was 25.5 years (SD = 10.3, Range: 14–62) and most persons were adults (75.1%, *n* = 560), female (95.2%, *n* = 710), and had at least one comorbid mental disorder (78.8%, *n* = 588), the most common of which were affective disorders (72.4%, *n* = 540), anxiety disorders (24.7%, *n* = 184), and personality disorders (17.6%, *n* = 131). Mean length of stay was 71.6 days (SD = 35.0, Range: 2–213).

The treatment at the hospital adheres to the German S3‐guidelines for the treatment of BN in terms of admission criteria, treatment elements, and therapy goals (Herpertz et al. 2019). Inpatient treatment is indicated when previous outpatient treatment has been unsuccessful or when other circumstances require a more intensive treatment setting (e.g., substantial mental and physical comorbidity, high illness severity). Persons received a cognitive behavior therapy‐oriented, multimodal BN treatment that included several treatment elements such as individual psychotherapy sessions, group therapy sessions, supervised meals, exercise therapy, meal preparation classes, body image exposure, nutrition counseling, food intake protocols, and clinical management of medical complications.

### 2.2 Measures

Body height was measured at admission and body weight was measured at admission and multiple times during persons' stay. However, weighing schedules differed between persons (e.g., some persons may have been weighed once a week and others were weighed every day). Thus, we converted each measurement to BMI (kg/m^2^) and then averaged the data for each week. In addition, we also calculated BMI at admission and discharge. As the sample included both adolescents and adults, we considered using age‐ and sex‐standardized BMI (computed with the R package *childsds*) but it was highly correlated with BMI at both admission (*r*_pb_ = 0.88) and discharge (*r*_pb_ = 0.86). Thus, we only used BMI to avoid redundancy and because it has a more straightforward interpretation. The Munich Eating and Feeding Disorder Questionnaire (Munich ED–Quest; Fichter et al. 2015) was part of the routine diagnostic assessment at admission and discharge and includes a question about highest body weight in kg (“What has been your highest weight since you have been fully grown?”). Responses to this question were converted to BMI and then weight suppression was calculated by subtracting BMI at admission from highest BMI.

The Munich ED–Quest has a subscale named *binge eating and vomiting* but this subscale also includes questions about associated features of binge eating and only produces one score for both binge eating and vomiting combined. As we were interested in testing separate and interactive effects of purging and binge eating, we thus selected two items from the Munich ED–Quest that specifically assess purging frequency and binge eating severity. Purging was assessed with the item “I vomited in order to avoid gaining weight,” which is answered on a five‐point scale from 0 = *never* to 4 = *very often*, referring to the last 3 months. We decided to only use this specific question for indicating purging as self‐induced vomiting is by far the most commonly used purging method in persons with BN (Dalle Grave et al. 2009; Dieffenbacher et al. 2025; Reba et al. 2005). Binge eating was assessed with the item “I experienced binge‐eating episodes where, in less than 2 h, I stuffed myself to a degree that others would have considered unusual,” which is answered on a five‐point scale from 0 = *not at all* to 4 = *very severely*, referring to the last 3 months.

### 2.3 Data Analyses

All analyses were conducted with R version 4.4.1 in RStudio version 2024.09.0 and for all inferential tests we considered effects as significant when *p* < 0.005, as has been recommended (Benjamin et al. 2018). Relationships between weight suppression, BMI, purging, and binge eating at admission were examined with robust percentage bend correlation coefficients (Mair and Wilcox 2020; Wilcox 1994) computed with the *WRS2* package version 1.1–6. Changes in BMI, purging, and binge eating from admission to discharge were tested with robust mixed models (Koller 2016) using the *robustlmm* package version 3.3–1. Specifically, a model was estimated for each dependent variable that included the fixed effect of time (admission vs. discharge) and a random intercept (i.e., person‐level random variability in scores at admission). As *robustlmm* does not produce parameter‐specific *p*‐values, we used the workaround described by Geniole et al. (2019) and fitted non‐robust models with the *lme4* package version 1.1–35.5 to obtain Satterthwaite‐approximated degrees of freedom. Two‐sided *p*‐values were then computed for the robust *t*‐values using the approximated degrees of freedom. Cohen's *d* (Cohen 1988) was computed as effect size for changes from admission to discharge with the *effectsize* package version 0.8.9.

As this was a naturalistic sample that included persons with different treatment durations (including persons that were discharged prematurely, persons that received regular treatment of several weeks, and persons that received an extended treatment of several months), sample size decreased with each treatment week. Thus, we had to decide how many weeks we would analyze to examine changes in BMI across treatment weeks. We decided to analyze the first 10 treatment weeks as there were still data of about half of the sample available in week 10 (49.5%, *n* = 369). Note, however, that BMI in week 10 does not represent BMI at discharge as most persons stayed shorter or longer than 10 weeks. Also note that computing mixed models uses maximum likelihood estimation, which does not remove cases with missing data (as would be done, e.g., in an analysis of variance by listwise deletion) and, thus, all models are based on the full sample of 746 cases.

**FIGURE 1:**
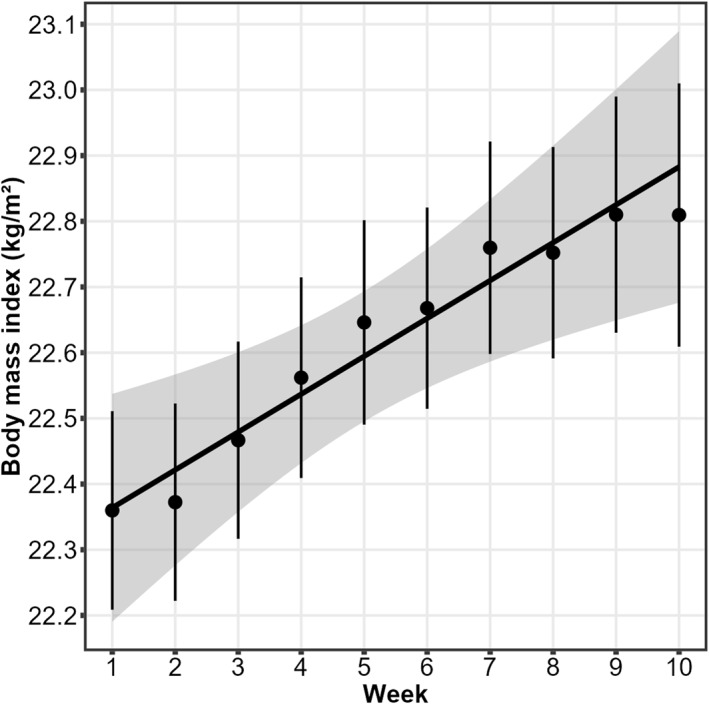
Mean body mass index as a function of treatment weeks. Error bars indicate standard errors of means. The bolded line represents a linear fit line and the grey‐shaded area its standard error.

When examining changes in BMI across treatment weeks (i.e., 10 weeks), we first tested whether changes were linear or non‐linear. For this, we created orthogonal polynomials (cf., Mirman 2014) of the week variable and estimated a robust mixed model that included fixed effects of both the linear term (first‐order polynomial) and the non‐linear term (second‐order polynomial) as well as a random intercept. However, only the linear term (estimate = 0.67, SE = 0.02, *p* < 0.001) but not the non‐linear term (estimate = − 0.03, SE = 0.01, *p* = 0.029) was significant, indicating that BMI changed linearly across weeks (Figure 1). Thus, we only examined effects on linear changes in the subsequent models. Specifically, we computed separate robust mixed models as described above that tested whether (1) weight suppression, (2) the interaction between weight suppression and BMI at admission, (3) purging at admission, (4) binge eating at admission, and (5) the interaction between purging and binge eating predicted changes in BMI across treatment weeks. We also tested whether adding length of stay, age, sex, and comorbidity (having vs. not having at least one comorbid mental disorder) as covariates to these models would change any of the effects of interest. The data and code with which all results can be reproduced can be accessed at https://osf.io/ghy9v.

## 3 Results

At admission, higher weight suppression weakly related to lower BMI and more frequent purging, which in turn weakly related to lower BMI (Table 1). Binge eating did not relate to weight suppression or BMI but weakly, positively related to purging (Table 1). From admission to discharge, BMI increased with a medium effect size and purging and binge eating decreased with large effect sizes (Table 1).

**TABLE 1:** Correlations between study variables at admission and descriptive statistics and test statistics for changes from admission to discharge.

	Percentage bend correlation coefficients at admission				Admission				Discharge				Estimate	SE	*t*	*df*	*p*	*d*
	1.	2.	3.	4.	*n*	*M*	SD	Range	*n*	*M*	SD	Range						
1. Weight suppression (kg/m^2^)	—	−0.25*	0.15*	0.04	746	3.73	3.90	0–31.9	—	—	—	—	—	—	—	—	—	—
2. Body mass index (kg/m^2^)	−0.25*	—	−0.12*	−0.08	746	22.3	4.17	14.5–43.8	746	22.9	3.80	16.2–42.5	0.62	0.04	14.4	746.0	< 0.001	0.49
3. Purging	0.15*	−0.12*	—	0.33*	737	3.00	1.44	0–4	610	0.39	0.94	0–4	−3.20	0.04	74.0	681.0	< 0.001	−1.64
4. Binge eating	0.04	−0.08	0.33*	—	743	3.27	1.14	0–4	624	0.33	0.90	0–4	−3.45	0.03	109.5	673.0	< 0.001	−2.23

*Note:* Estimate, SE and *t* were obtained with robust models, Satterthwaite‐approximated degrees of freedom were obtained with similar non‐robust models to compute *p*‐values using Geniole et al.'s (2019) workaround.

^*^ *p* < 0.001.

**FIGURE 2:**
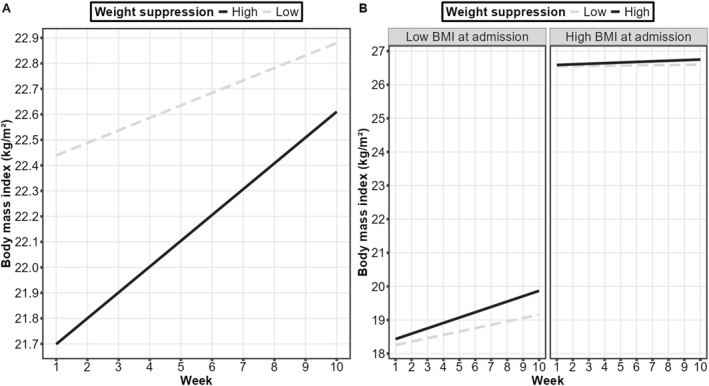
Simple slopes for probing the interaction effects (A) week × weight suppression and (B) week × weight suppression × body mass index at admission when predicting changes in body mass index. The denotations “high” and “low” refer to +/− one standard deviation from the mean of the moderator variables. Note that this does not mean that the data were categorized into groups: all analyses were run with continuous variables and this depiction only serves the purpose of visualizing the interaction effects.

A significant week × weight suppression interaction (Table 2) indicated that weight suppression moderated BMI changes across weeks: higher weight suppression predicted larger weight gain (Figure 2A). However, a significant week × weight suppression × BMI interaction (Table 2) indicated that weight suppression and BMI at admission interactively moderated BMI changes across weeks: the effect of higher weight suppression being predictive of a steeper weight gain slope was particularly pronounced in persons with a relatively low BMI at admission (Figure 2B).

**TABLE 2:** Coefficients and test statistics for independent variables (intercepts not shown) predicting body mass index (BMI).

Independent variables	Estimate	SE	*t*	*df*	*p*
Weight suppression model					
Week	0.05	0.002	22.5	5269.2	< 0.001
Weight suppression	−0.10	0.03	3.53	752.3	< 0.001
Week × weight suppression	0.01	0.0004	16.5	5269.4	< 0.001
Weight suppression × BMI at admission model					
Week	0.31	0.01	28.1	5349.1	< 0.001
Weight suppression	0.04	0.03	1.21	974.0	0.227
BMI at admission	0.99	0.01	143.7	971.9	< 0.001
Week × weight suppression	0.02	0.002	9.06	5345.8	< 0.001
Week × BMI at admission	−0.01	0.001	24.4	5348.0	< 0.001
Weight suppression × BMI at admission	−0.001	0.001	0.96	973.2	0.340
Week × weight suppression × BMI at admission	−0.001	0.0001	7.23	5344.7	< 0.001
Purging model					
Week	0.04	0.004	9.88	5208.4	< 0.001
Purging	−0.24	0.08	3.09	743.3	0.002
Week × purging	0.01	0.001	10.1	5208.3	< 0.001
Binge eating model					
Week	0.10	0.006	18.2	5245.0	< 0.001
Binge eating	−0.16	0.10	1.61	750.1	0.108
Week × binge eating	−0.01	0.002	5.17	5244.7	< 0.001
Purging × binge eating model					
Week	0.12	0.01	11.9	5184.1	< 0.001
Purging	−0.003	0.21	0.02	740.9	0.988
Binge eating	0.10	0.18	0.56	740.7	0.577
Week × purging	−0.01	0.004	1.44	5183.5	0.150
Week × binge eating	−0.03	0.003	8.94	5183.6	< 0.001
Purging × binge eating	−0.07	0.06	1.10	740.7	0.271
Week × purging × binge eating	0.01	0.001	5.75	5183.2	< 0.001

*Note:* Estimate, SE and *t* were obtained with robust models, Satterthwaite‐approximated degrees of freedom were obtained with similar non‐robust models to compute *p*‐values using Geniole et al.’s (2019) workaround.

**FIGURE 3:**
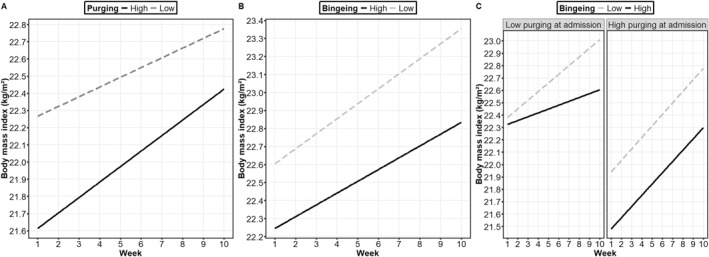
Simple slopes for probing the interaction effects (A) week × purging, (B) week × binge eating, and (C) week × purging × binge eating when predicting changes in body mass index. The denotations “high” and “low” refer to +/− one standard deviation from the mean of the moderator variables. Note that this does not mean that the data were categorized into groups: all analyses were run with continuous variables and this depiction only serves the purpose of visualizing the interaction effects.

A significant week × purging interaction (Table 2) indicated that purging at admission moderated BMI changes across weeks: more frequent purging at admission predicted larger weight gain (Figure 3A). A significant week × binge eating interaction (Table 2) indicated that binge eating at admission moderated BMI changes across weeks: less severe binge eating at admission predicted larger weight gain (Figure 3B). However, a significant week × purging × binge eating interaction (Table 2) indicated that purging and binge eating at admission interactively moderated BMI changes across weeks: at frequent purging, binge eating severity did not affect weight gain slopes but at infrequent purging, higher binge eating severity attenuated weight gain (Figure 3C). All effects of interest (i.e., the highest‐order interaction effects) in all mixed models were unaffected by including length of stay, age, sex, and comorbidity as covariates (i.e., were still significant at *p* < 0.001).^1^

## 4 Discussion

The current study examined weight change during inpatient treatment in persons with BN. On average, persons moderately gained weight from admission to discharge, that is, weight gain was statistically significant, but effect size was medium. In contrast to findings about a non‐linear weight trajectory in persons with anorexia nervosa (Jennings et al. 2018; Meule et al. 2022; Vansteelandt et al. 2010), weight gain was linear across treatment weeks. This suggests that future studies on weight change during treatment in persons with BN may rely on BMI assessed at the beginning and end of treatment without losing much information. That is, as weight increases linearly during treatment, measuring it at two time points suffices for appropriately modeling these changes.

Higher weight suppression at admission predicted larger weight gain, which is in line with other studies (Carter et al. 2008; Herzog et al. 2010; Hessler et al. 2018; Lowe, Davis, et al. 2006b). Yet, this effect was moderated by BMI at admission such that the effect of weight suppression on weight gain was larger in persons with a relatively low BMI at admission. While this is the first study that reports such an interactive effect in persons with BN, it dovetails with similar interaction effects that have been reported in persons with anorexia nervosa and other samples (Berner et al. 2013; Call et al. 2021; Meule et al. 2022; Witt et al. 2014). This indicates that those with a relatively low BMI and high weight suppression in combination are at greatest risk of gaining weight during treatment.

More frequent purging at admission predicted larger weight gain as well. As purging weakly related to higher weight suppression and to lower BMI at admission and strongly decreased from admission to discharge, this suggests that more frequent purging had successfully led to larger weight loss before treatment (or was used to maintain that low body weight) but this weight is then partially regained during treatment when persons abstain from purging. In contrast, lower binge eating severity at admission predicted larger weight gain, the exact mechanisms of which require investigation in future studies. Of note, binge eating and purging were only weakly correlated at admission, indicating that they do not always co‐occur. In line with our hypotheses derived from this, binge eating and purging interacted in the prediction of weight change. Probing the nature of this interaction revealed that binge eating severity did not moderate weight gain in persons with frequent purging but high binge eating severity attenuated weight gain in persons with infrequent purging. Again, the exact mechanisms of this require future investigation but this result further supports the notion that examining interactive effects between binge eating and purging may be a fruitful future avenue in research about associated features of BN such as general psychopathology, life satisfaction, and body weight (Dieffenbacher et al. 2025).

### 4.1 Clinical Implications

The current findings have implications for clinical practice. First, while they show that persons with BN linearly gain weight during inpatient treatment, they also show that this weight gain is moderate. Specifically, average weight gain was less than one BMI point while average weight suppression was more than three BMI points. Thus, therapists could use this information by discussing with patients that they may not necessarily regain their highest past weight or even more when they refrain from purging. Second, the fact there will likely be some weight regain—particularly when frequent purging has induced a large weight loss—could be used for psychoeducation, highlighting the biological pressures to regain lost weight, as has been detailed by others (Juarascio et al. 2018). Finally, addressing these topics early in treatment may improve treatment adherence when they are combined with an Acceptance and Commitment Therapy approach, which teaches patients to obtain psychological distance from distressing internal experiences, to clarify overarching personal values, to create goals that can help patients live a more fulfilling life, and to increase willingness to experience negative internal experiences in the service of valued behavior. This may then facilitate that patients can accept if weight gain actually occurs, thereby preventing the emergence of negative feelings, which would otherwise may again trigger the vicious cycle of binge eating and purging (Bavi et al. 2025; Manlick et al. 2013).

### 4.2 Limitations

Interpretation of the current findings is limited to persons with BN receiving inpatient treatment in Germany and may, thus, not translate to persons with BN in other treatment settings or to countries with different healthcare systems. Furthermore, progress has been made in the study of weight suppression with different calculation methods having been proposed (Schaumberg et al. 2016; Singh et al. 2021). Thus, future studies may determine whether results of the current study replicate using these approaches (e.g., using developmental weight suppression). Moreover, findings are limited to weight change during treatment but a future avenue would be to also examine weight change and its predictors after treatment. In addition, the current analyses relied on BMI as a proxy for body fat mass and although BMI is a good estimate of body fat mass, it is not optimal (Romero‐Corral et al. 2008). However, BMI and percent body fat are indeed almost perfectly correlated (around *r* = 0.9) in young women (Meule and Platte 2018; Romero‐Corral et al. 2008). As most persons in the current sample were young women, we assume that findings would be congruent if we had data on percent body fat available. Finally, binge eating and purging were assessed with two single items based on self‐report, which may not be as reliable as multi‐item measures or external assessment and, thus, the current results require replication in studies using other assessment techniques such as diagnostic interviews.

### 4.3 Conclusion

The current study replicates and extends earlier reports by showing that both weight suppression and current BMI in combination and both binge eating and purging in combination are predictive of weight gain during treatment in persons with BN. While average weight gain during inpatient treatment is moderate (less than one BMI unit), weight gain is larger in certain subgroups (e.g., those with low BMI and high weight suppression). As fear of gaining weight has driven these persons to engage in purging in the first place, this fear then becomes true during treatment. As this threatens persons' motivation to abstain from purging behaviors, therapists should address the likelihood of gaining weight as a result of reducing self‐induced vomiting early in treatment.

## Ethics Statement

According to the ethics committee of the LMU Munich, retrospective analyses of completely anonymized data are exempt from requiring ethical approval.

## Consent

According to the ethics committee of the LMU Munich, retrospective analyses of completely anonymized data are exempt from requiring informed consent.

## Conflicts of Interest

The authors declare no conflicts of interest.

## Supporting information

Supporting Information S1

## Data Availability

The data and code with which all results can be reproduced can be accessed at https://osf.io/ghy9v.

## Endnotes

^1^
One of the reviewers asked if results were different for adolescents and adults. To answer this question, we tested whether age moderated any effects of interest (i.e., the highest‐order interaction effects). The code for these analyses can be found here https://osf.io/ghy9v and results are described in the Supporting Information S1.
