# Supplementary material for: Weight Suppression, Binge Eating, and Purging as Predictors of Weight Gain During Inpatient Treatment in Persons With Bulimia Nervosa
Source: Eur Eat Disord Rev. 2025 Apr 7;33(5):941–9. doi: 10.1002/erv.3197 (PMC12319125; doi:10.1002/erv.3197)
Supplement: Supplementary file 1 — Supporting Information S1 [file ERV-33-941-s001.pdf]

# Supplemental material

One of the reviewers asked if results were different in adolescents and adults. Thus, we re-ran all models by testing whether age moderated any effects of interest. The data and code for these analyses can be accessed at https://osf.io/ghy9v. For the weight suppression model, there was a significant week × weight suppression × age interaction effect ($b$ = –0.0004, $SE$ = 0.00004, $p$ < .001), indicated that the week × weight suppression interaction differed as a function of age. Figure S1A plots this interaction effect for adolescents and adults. For the weight suppression × BMI at admission model, the week × weight suppression × BMI at admission × age interaction effect was not significant ($b$ = –0.00003, $SE$ = 0.00001, $p$ = .007), indicating that the week × weight suppression × BMI at admission interaction did not differ as a function of age. For the purging model, there was a significant week × purging × age interaction effect ($b$ = –0.001, $SE$ = 0.0001, $p$ < .001), indicated that the week × purging interaction differed as a function of age. Figure S1B plots this interaction effect for adolescents and adults.

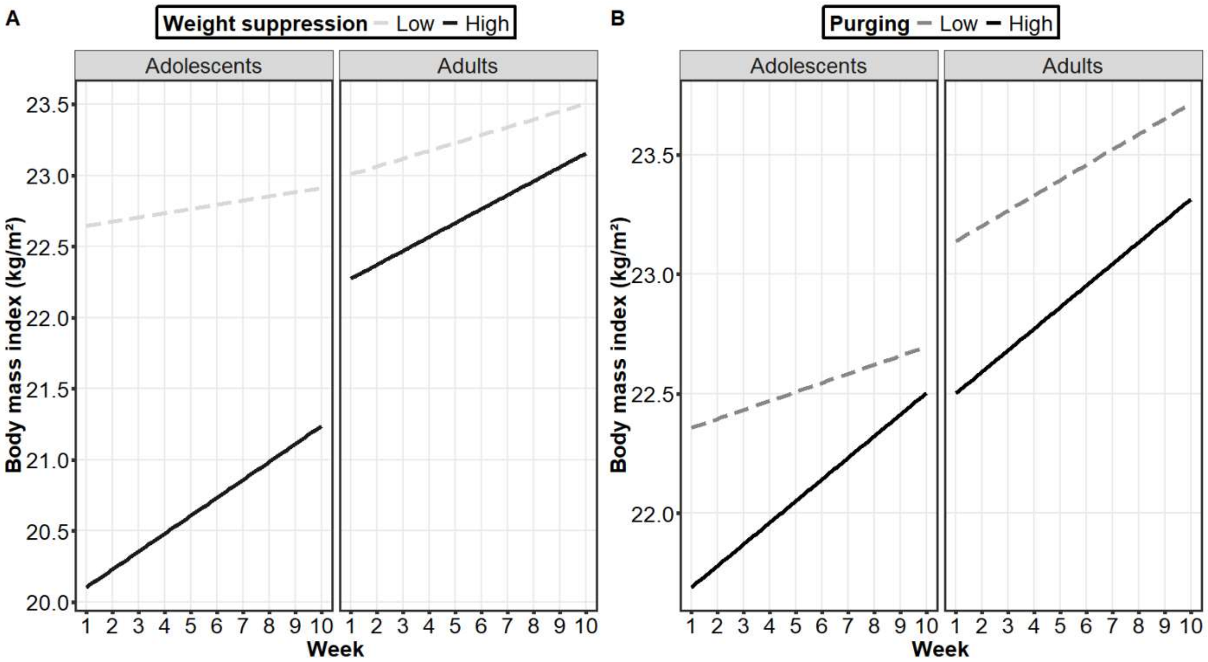


*Figure S1*. Simple slopes for probing the interaction effects (A) week × weight suppression × age and (B) week × purging × age when predicting changes in body mass index. The denotations “high” and “low” refer to +/– one standard deviation from the mean of the moderator variables.

For the binge eating model, the week × binge eating × age interaction effect was not significant ($b$ = 0.0003, $SE$ = 0.0002, $p$ = .030), indicating that the week × binge eating interaction did not differ as a function of age. For the purging × binge eating model, there was a significant week × purging × binge eating × age interaction effect ($b$ = 0.0005, $SE$ = 0.0001, $p$ < .001), indicated that the week × purging × binge eating interaction differed as a function of age. Figure S2A plots this interaction effect for adolescents and Figure S2B plots this interaction effect for adults.

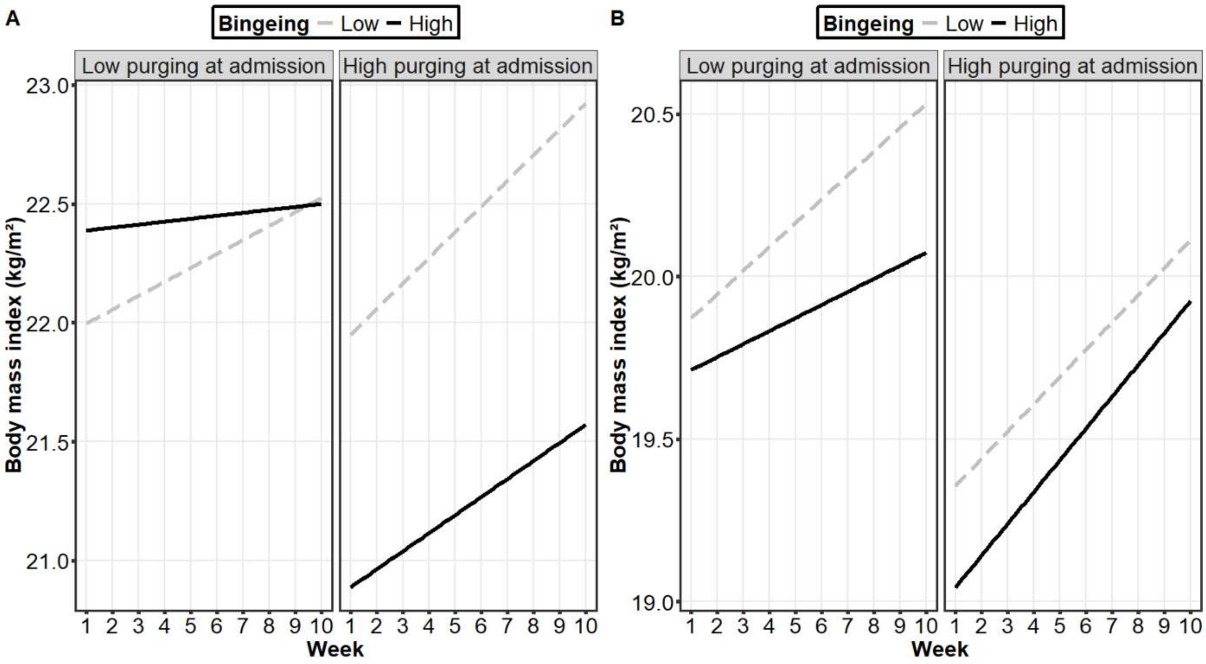


*Figure S2*. Simple slopes for probing the interaction effect week × purging × binge eating × age when predicting changes in body mass index. Panel A displays the interaction week × purging × binge eating for adolescents and panel B displays the interaction week × purging × binge eating for adults. The denotations "high" and "low" refer to +/– one standard deviation from the mean of the moderator variables.
